# Effects of dietary L‐arginine supplementation to early pregnant mares on conceptus diameter—Preliminary findings

**DOI:** 10.1111/rda.13422

**Published:** 2019-03-08

**Authors:** Jörg Aurich, Martin Köhne, Manuela Wulf, Christina Nagel, Elisabeth Beythien, Camille Gautier, Jürgen Zentek, Christine Aurich

**Affiliations:** ^1^ Department of Small Animals and Horses, Obstetrics, Gynaecology and Andrology Vetmeduni Vienna Vienna Austria; ^2^ Graf Lehndorff Institute for Equine Science Vetmeduni Vienna Neustadt (Dosse) Germany; ^3^ Institute for Animal Nutrition Free University Berlin Berlin Germany; ^4^ Department of Small Animals and Horses, Artificial Insemination and Embryo Transfer Vetmeduni Vienna Vienna Austria

**Keywords:** arginine, embryo, foetus, horse

## Abstract

The importance of the amino acid L‐arginine (ARG) for conceptus growth and litter size has been demonstrated in various species. L‐arginine is part of embryo‐derived polyamines, a substrate for nitric oxide synthase and stimulates protein synthesis by the embryo. In the present study, we have investigated whether dietary L‐arginine supplementation stimulates early conceptus growth in mares. Warmblood mares with singleton pregnancies received either an arginine‐supplemented diet (approximately 0.0125% of body weight, *n* = 12) or a control diet (*n* = 11) from days 15 to 45 after ovulation. Diameter of the embryonic vesicle (days 14, 17, 20 of pregnancy) and size of the embryo respective foetus (length and maximal diameter, days 25–45 of pregnancy at 5‐day intervals) were determined by transrectal ultrasound. At foaling, weight and size of the foal and the placenta were determined. Blood for determination of equine chorionic gonadotrophin (eCG) and progestin concentrations was collected repeatedly. Neither eCG nor progestin concentration in plasma of mares differed between groups at any time. No effects of arginine treatment on diameter of the embryonic vesicle between days 14 and 20 of pregnancy were detected. Diameter of the embryo/foetus on days 40 to 45 of pregnancy strongly tended to be enhanced by arginine supplementation (*p* = 0.06). Weight and size of neither the foal nor placenta at birth differed between groups. In conclusion, L‐arginine supplementation was without negative effects on early equine embryos and may support embryonic growth at the beginning of placentation.

## 1 INTRODUCTION

In the horse, embryonic losses can substantially reduce fertility (Adams, Kastelic, Bergfelt, & Ginther, 1987; Morris & Allen, 2002). Delayed development of the conceptus is a major cause of early embryonic death in mares (Adams et al., 1987; Bergfelt, Woods, & Ginther, 1992; Ginther, Bergfelt, Leith, & Scraba, 1985; Woods, Baker, Hillman, & Schlafer, 1985). Mares with a history of early embryonic loss are often routinely treated with the progestin altrenogest (Allen, 2001). At the beginning of placentation between days 30 and 40 of pregnancy, size of the conceptus is smaller in older compared to young mares (Willmann, Schuler, Hoffmann, Parvizi, & Aurich, 2011). Treatment with altrenogest stimulated embryonic growth in older mares and thus accelerated a delayed development (Willmann et al., 2011). Treatment with human chorionic gonadotrophin before ovulation increased progestin concentration in early equine pregnancies and thus might reduce embryonic loss (Köhne, Kuhl, Ille, Erber, & Aurich, 2014).

Progestins in some countries are not licensed for long‐term treatment of pregnant mares. There is thus a research interest in non‐hormonal alternatives to enhance embryonic development in horses. In several mammalian species, positive effects of the amino acid L‐arginine on both embryo development around the time of placentation and foetal growth at later stages of pregnancy have been documented. L‐Arginine is a precursor of important metabolites including nitric oxide (NO) and polyamines. Along different growth factors, NO and polyamines are crucial for angiogenesis, embryogenesis, placental and foetal growth, uteroplacental blood flow, and transfer of nutrients from the mother to its foetuses, as well as foetal and placental growth and development. Additionally, arginine activates the mechanistic target of rapamycin (mTOR) cell signalling pathway to stimulate protein synthesis in the placenta, uterus and foetus (Bazer, Wu, & Johnson, 2017; Wu, Bazer, Cudd, Meininger, & Spencer, 2004; Wu et al., 2013). Arginine also has been demonstrated to enhance maternal pregnancy recognition in sheep via increased embryonic interferon tau production (Wang et al., 2015).

In pigs, dietary L‐arginine supplementation for different time periods before day 30 of gestation increased the number of piglets born alive and total litter weight as well as placental weight (Bérard & Bee, 2010; Li et al., 2010; Li et al.,^2014^; Mateo et al., 2007). Also in other species such as sheep, mice and rats, arginine supplementation had positive effects on foetal growth (Wu et al., 2013). Arginine supplementation at later stages of pregnancy also increased average birth weight of life‐born piglets by enhancing placental vascular function and promoting nutrient supply to the foetuses (Wu et al., 2012), reduced intra‐uterine growth retardation in prolific ewes carrying multiple foetuses (Lassala et al., 2011) and improved growth and development of human IUGR foetuses (Shen & Hua, 2011).

Only few reports exist on arginine feed supplementation and reproductive function in horses. Dietary arginine supplementation in mares during the last 4 months of pregnancy increased foal weight at birth compared to control animals (Chavatte‐Palmer et al., 2018). Feeding arginine during the last days of pregnancy accelerated expulsion of fluid from the post‐partum uterus (Kelley, Warren, & Mortensen, 2013) and reduced the incidence of mating‐induced endometritis when mares were bred again after foaling (Mesa, Warren, Sheehan, Kelley, & Mortensen, 2015). Supplementing the feed of mares with arginine for 3 weeks before the expected date of foaling shortened gestation length and increased uterine arterial blood flow before and after parturition (Mortensen, Kelley, & Warren, 2011). In another study, arginine feed supplementation to cyclic mares before embryo transfer increased the size and tended to increase perfusion of the ovulatory follicle, but had no effect on embryo recovery (Kelley, LeBlanc, Warren, & Mortensen, 2014). To the best of our knowledge, no information of the effects of arginine feed supplementation in early equine pregnancies is available. Therefore, in this study, effects of arginine feeding in early equine pregnancy on conceptus development and pregnancy outcome were investigated. We hypothesized that arginine stimulates conceptus development and synthesis of equine chorionic gonadotrophin (eCG) from the endometrial cups and may therefore reduce the incidence of early pregnancy loss in the horse.

## 2 MATERIALS AND METHODS

The study was approved by the competent authority for animal experimentation in Brandenburg State, Germany (*Landesamt für Arbeitsschutz, Verbraucherschutz und Gesundheit*, license number 2347‐8‐2016).

### 2.1 Animals

A total of 22 warmblood broodmares and their newborn foals at the Brandenburg State Stud in Neustadt (Dosse), Germany, were included in the study. At the beginning of the study, mares were between 3 and 17 years old (8.0 ± 4.6 years, mean ± *SD*) and weighed between 466 and 639 kg (570 ± 45 kg, mean ± *SD*). Further information on the mares is given in Table 1. From October to April (stable period), the mares were housed in group stables (5–8 horses per group) with daily access to an outdoor paddock for 4 hr. During the stable period, mares were fed 200 g whole oats per 100 kg bodyweight twice daily (at 6:00 and 16:15). Hay was fed twice daily (8 kg/mare) but uptake of hay was not controlled. Mineral supplements and water were freely available at all times. From May to September (pasture period), mares had daily access to pasture grazing but were kept in group stables overnight. During the pasture period, the mares were fed 100 to 200 g oats/100 kg bodyweight once daily (at 6:00) depending on their body condition and hay was freely available during the time spent in the stable (20:00–8:00). One to two weeks before the calculated day of parturition, housing was changed to box stalls but the mares had daily access to an outdoor paddock together with group mates. In the foaling stable, mares were observed 24 hr per day. Birth of the foals was observed, but no obstetrical intervention was needed. One foal, however, was born dead on 327 days of gestation and one foal born on day 340 of gestation had to be euthanized after birth because of severely contracted flexor tendons in both hindlimbs. These two foals were considered pathological cases and not included in the analysis. Thus, 22 mares and their foetuses but only 20 foals were included in the analysis.

**Table 1:** Age, weight and height at withers at the start of the experiment and day of ovulation (within year) leading to pregnancy in mares treated either with arginine (*n* = 12) or left untreated as controls (*n* = 10), data are means ± *SEM*

Parameter	Arginine	Control	*p*‐value
Age (years)			
Mean ± *SEM*	6.8 ± 1.0	8.4 ± 1.5	0.364
Range (min–max)	3–15	3–16	
Primiparous mares	3/12	3/10	
Weight (kg)			
Mean ± *SEM*	569 ± 14	572 ± 14	0.884
Range (min–max)	466–640	497–661	
Height at withers (cm)			
Mean ± *SEM*	169 ± 1	167 ± 1	0.172
Range (min–max)	164–175	162–170	
Ovulation (day after 31.12.)			
Mean ± *SEM*	120 ± 8	116 ± 12	0.764
Range (min–max)	72–163	72–162	

### 2.2 Experimental procedures

All mares were bred by artificial insemination. When they showed signs of oestrus or were at the expected time of first post‐partum oestrus (approximately 6 days after foaling), their ovaries were examined for the presence of follicles and the uterus for the presence of endometrial oedema by rectal palpation and scanning with a 7.0 MHz linear ultrasound scanner (DP‐6600Vet, Mindray, Shenzhen, China). When a moderate or severe uterine oedema and a pre‐ovulatory follicle of 35 mm diameter were detected, mares were artificially inseminated with fresh or cooled‐stored semen from stallions chosen by the stud farm director. If required for management reasons (limited availability of semen on weekends and public holidays), ovulation was induced with human chorionic gonadotrophin (hCG, 1,500 IU i.v., Ovogest 300, MSD Animal Health, Unterschleissheim, Germany) as soon as the pre‐ovulatory follicle reached a size of 35 mm in diameter and endometrial oedema was detectable. The day when the pre‐ovulatory follicle was no longer detectable on ultrasound examination was defined as the day of ovulation. All ovulations leading to a pregnancy occurred between 13 March and 11 June of the same year.

On day 14 after ovulation, mares were checked for the presence of a pregnancy by transrectal ultrasonography. Mares with singleton pregnancies were assigned either to the experimental (group ARG, *n* = 12) or control group (group CON, *n* = 10). Mares of group ARG were fed arginine (Ajinomoto Omnichem, Mont‐Saint‐Guibert, Belgium; 75 g, corresponding to 0.0125% of bodyweight in a mare with 600 kg bodyweight) once daily with their morning feed until day 45 of pregnancy while CON mares received their normal feed ration. Arginine was incorporated into pellets consisting of 70% oatmeal, 15% alfalfa meal and 15% dry wood shavings and was readily consumed by the mares. Complete consumption of arginine‐containing pellets was ascertained by controlled individual feeding of mares. In order to reduce competition of arginine with lysine for absorption at the intestinal site, we slightly reduced the dose of arginine (from 100 g to 75 g/mare) compared to previous studies (Kelley et al., 2014; Mortensen et al., 2011). Based on arginine content in oats and hay (Hackl, Hoven, Zickl, Spona, & Zentek, 2006), the normal feed ration fed to mares of the ARG and CON groups per day contained approximately 42 g arginine and 34 g lysine. Assignment of mares to groups was basically made in alternating order but adjusted to avoid group differences with regard to age and number of lactating and non‐lactating mares per group (see Table 1).

### 2.3 Development of pregnancy

Conceptus development was assessed by transrectal ultrasonography on days 14, 17, 20, 25, 30, 35, 40 and 45 of pregnancy as described previously (Willmann et al., 2011). Using a 7.0 MHz linear ultrasound scanner (DP‐50Vet, Mindray), the diameter of the embryonic vesicle was determined on days 14, 17 and 20 of pregnancy and length (maximal size along the longitudinal axis) and diameter (maximal size perpendicular to the longitudinal axis) of the embryo proper were measured on days 25 to 45 with the electronic callipers of the ultrasound system as described (Köhne et al., 2014; Willmann et al., 2011). The examiner performing ultrasonography was not aware of the treatment the mares received. Weight of the mares was determined at monthly intervals throughout pregnancy. Variability of conceptus measurement was validated with two independent observers on other horses of the same breed and pregnancy stage (*n* = 9). Inter‐observer variation was 4.4% for conceptus length (3.1 ± 0.5 cm, mean ± *SD*) and 7.9% for conceptus diameter (1.6 ± 0.3 cm).

After birth and shedding of the placenta, placental weight was recorded and the placental surface area was measured as described (Allen et al., 2002; Beythien, Aurich, Wulf, & Aurich, 2017). In the foals (group ARG *n* = 11, group CON *n* = 9), the following parameters were determined between 6 and 12 hr after birth as described (Allen, Wilsher, Tiplady, & Butterfield, 2004; Beythien et al., 2017): body weight, height at withers, chest circumference, distance from fetlock to carpal joint, distance from carpal joint to elbow, crown‐rump length and poll‐to‐nose length.

### 2.4 Endocrine analyses

Blood samples for hormone analysis were taken into vacutainer tubes (Vacuette, Greiner, Frickenhausen, Germany) from one jugular vein at daily intervals from days 14 to 21, at five‐day intervals from day 25 to 45 and on day 60 of pregnancy.

Progesterone in plasma of mares was determined by enzyme‐linked immunosorbent assay (ADI‐900‐011; Assay Designs, Ann Arbor, MI, USA) as described previously and validated for equine plasma in our laboratory (Nagel et al., 2012). The antiserum cross‐reacts 100% with 5α‐pregnane‐3,20‐dione, thus besides progesterone measuring accurately the most important pregnancy‐specific equine progestin. According to the manufacturer, cross‐reactivity is 3.5% with 17‐OH‐progesterone and <1% for other steroids tested. The intra‐assay coefficient of variation was 7.5%and 9.0%, respectively, for low and medium‐range reference plasma, and the interassay coefficient of variation was 10.1% and 8.5%, respectively, for the low and medium‐range reference plasma. The minimal detectable concentration of the assay was 8.6 pg/ml.

Equine chorionic gonadotrophin (eCG) in plasma was determined by enzyme‐linked immunosorbent assay (DE1298; Demeditec, Kiel, Germany). In our laboratory, serial dilution of plasma from a pregnant mare showed strict parallelity to the standard curve and recovery of standard added to plasma was 85%. The interassay coefficient of variation was 9.2% and 7.3%, respectively, for low and medium‐range reference plasma (*n* = 6 microtitre plates), and the interassay coefficient of variation calculated from the duplicate measurements of all plasma samples was 8.3%. The minimal detectable concentration of the assay calculated as two standard deviations from zero binding was 0.25 mIU/ml. According to the manufacturer's information, equine FSH and LH at concentrations of 50 ng/ml produced a colour intensity equivalent to 0.92 and 0.51 IU of eCG/ml, respectively. Samples were diluted 1:10 when low eCG concentrations were to be expected (days 30 and 35 of pregnancy) and at least 1:100 for all other samples, thus minimizing effects of cross‐reactivity with FSH and LH.

### 2.5 Statistical analysis

Statistical comparisons were made with the statistics software SPSS version 20.0 (SPSS‐IBM, Armonck, NY, USA). All data were normally distributed (Kolmogorov–Smirnov test). Parameters determined repeatedly were analysed by ANOVA with a general linear model (GLM) for repeated measures with time as within‐subject factor and group as between‐subject factor. Parameters determined only once were compared by one‐way ANOVA. All values given are means ± standard error of mean (*SEM*).

## 3 RESULTS

**Figure 1:**
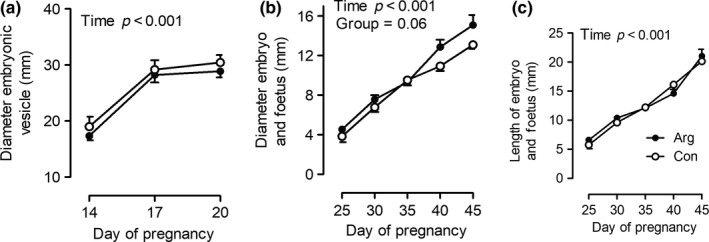
(a) Diameter of the embryonic vesicle from days 14 to 20 of pregnancy and (b) diameter and (c) length of the embryo proper and foetus from days 25 to 45 of pregnancy in mares either fed arginine as a feed supplement from days 14 to 45 of pregnancy (group ARG, ●, *n* = 12) or left untreated (group CON, ○, *n* = 10), significant differences are indicated in the figures

The diameter of the embryonic vesicle increased significantly from days 14 to 17 after ovulation, showed no major increase thereafter until day 20 and did not differ between mares of groups ARG and CON (Figure 1a). The diameter and length of the embryo and foetus increased constantly between days 25 and 45 of pregnancy (*p* < 0.001, Figures 1b, c). For the diameter of the embryo proper and foetus, a group difference was nearly reached (*p* = 0.06) and foetal diameter strongly tended to be bigger in group ARG compared to CON (Figure 1b). Post hoc power analysis for repeated measures ANOVA of foetal diameter revealed a statistical power (1‐*β* error probability) of 0.8.

**Figure 2:**
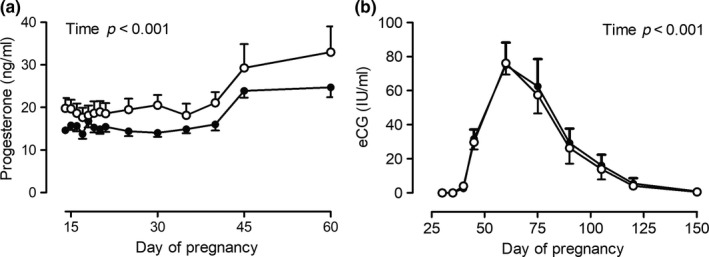
(a) Progesterone concentration in plasma from days 14 to 60 of pregnancy and (b) eCG concentration in plasma from days 30 to 150 of pregnancy in mares either fed arginine as a feed supplement from days 14 to 45 of pregnancy (group ARG, ●, *n* = 12) or left untreated (group CON, ○, *n* = 10), significant differences are indicated in the figure

Plasma progesterone concentration during the treatment period and for 15 days thereafter did not differ between ARG and CON mares, but in mares of both groups increased after day 45 of pregnancy (*p* < 0.001, Figure 2a). The concentration of eCG in plasma of mares was at the lower detection limit of the eCG assay on days 30 and 35 of pregnancy, increased thereafter to a peak on day 60 and declined gradually until day 150 (*p* < 0.001 over time). The concentration of eCG did not differ between groups, and curves for ARG and CON mares were close to identical (Figure 2b).

**Figure 3:**
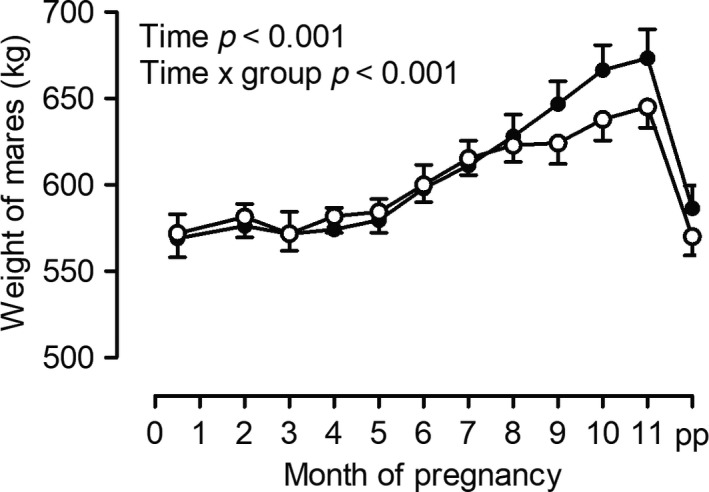
Weight of mare either fed arginine as a feed supplement from days 14 to 45 (group ARG, ●, *n* = 12) or left untreated (group CON, ○, *n* = 10) throughout pregnancy and one day after foaling (pp), significant differences are indicated in the figure

As expected, weight of mares clearly increased throughout the second half of pregnancy (*p* < 0.001, Figure 3). This increase was more pronounced in mares fed arginine from days 14 to 45 of pregnancy (ARG) than in control mares (CON, time × group *p* < 0.001). At birth, neither gestation length nor placental weight and surface or any of the size parameters determined in the newborn foals differed significantly between groups (Table 2).

**Table 2:** Characteristics of the placenta and newborn foal of mares treated either with arginine (mares *n* = 12, foals *n* = 11) or left untreated as controls (mares *n* = 10, foals *n* = 9), data are means ± *SEM*

	Arginine	Control
Gestation length (days)	337 ± 2	337 ± 3
Placental weight (g)	7,288 ± 474	6,500 ± 313
Placental surface (cm^2^)	13,225 ± 511	13,100 ± 366
Foal weight (kg)	56 ± 2	56 ± 1
Height at withers (cm)	104 ± 2	107 ± 1
Distance elbow—carpus (cm)	32.1 ± 0.8	32.2 ± 0.6
Distance carpus—fetlock (cm)	27.1 ± 0.4	27.1 ± 0.5
Circumference cannon bone (cm)	13.6 ± 0.2	13.5 ± 0.2
Length nose—poll (cm)	42.9 ± 0.5	42.3 ± 0.5
Length poll—tail (cm)	98 ± 2	97 ± 2
Circumference of chest (cm)	86 ± 1	86 ± 1

Note

No significant differences between groups.

## 4 DISCUSSION

This study provides preliminary evidence that supplementing the feed of mares in early pregnancy with the amino acid L‐arginine may increase conceptus growth. This effect was relatively small and restricted to the early phase of placentation which in horses occurs around day 40 of pregnancy (Stewart & Allen, 1995). To what extent, L‐arginine supplementation enhanced embryonic growth to a point that improves conceptus survival thus remains open. In early pregnant mares with an experimentally reduced luteal function, lysine and isoleucine but not arginine content in uterine fluid was reduced (Beyer et al., 2019), adding further evidence that arginine may be less important during early pregnancy in horses than in certain other species.

Although less clear, results of the present study are nevertheless largely in agreement with results obtained in pigs (Bérard & Bee, 2010; Li et al.,^2010^; Li et al.,^2014^; Mateo et al., 2007). In the polytocous porcine species, effects of dietary L‐arginine supplementation in early pregnancy were more evident than in horses and mainly achieved by increasing the number of foetuses through enhanced placental function, resulting in a higher total litter weight and total placental weight than in control animals.

Our study extends findings in the polytocous pig to the monotocous equine species. In the pig, more embryos in early pregnancy finally resulted in more piglets born although treatment was discontinued at approximately 30 days of pregnancy (Bérard & Bee, 2010; Li et al., 2010; Mateo et al., 2007). In contrast, effects of arginine on conceptus growth at the beginning of placentation in monotocous species like the horse are unlikely to persist until foaling. It is therefore not surprising that weight and size neither of the foals nor the placenta differed between arginine‐supplemented and control pregnancies at birth. However, the present study does not allow to conclude whether—in agreement with the situation in pigs—arginine treatment in horses might rescue early pregnancies that otherwise would have undergone early conceptus death. Death of the conceptus before placentation contributes substantially to subfertility in mares, and reduced endometrial function is considered an important underlying cause (Adams et al., 1987; Bergfelt et al., 1992; Ginther et al., 1985; Woods et al., 1985). The mares of our present study were all characterized by good fertility, and only few older, potentially less fertile mares were included.

Several studies have demonstrated a crucial role for NO in enhancing blood flow during ovine pregnancy (Gardner, Powlson, & Giussani, 2001; McCrabb & Harding, 1996). Both NO and polyamines play key roles in angiogenesis, which is a critical event during placental growth and foetal development (Bazer et al., 2017; Wu et al.,^2004^; Wu et al.,^2013^). Formation of the endometrial cups by invasion of the endometrium from chorionic girdle cells marks the beginning of placentation in the horse (Antczak, Mestre, Wilsher, & Allen, 2013). Therefore, we used eCG as a marker for placentation and hypothesized that feed supplementation with L‐arginine in early pregnancy may increase eCG synthesis and release from the endometrial cups. The concentration of eCG, however, was close to identical in arginine‐treated and control mares throughout the physiological phase of eCG secretion. Arginine thus is without effect on eCG synthesis and release and does not exert an influence on equine pregnancy via eCG. Consequently, also progesterone concentration in plasma of mares increased in response to endogenous eCG but was not affected by arginine treatment.

In sheep and pigs, arginine treatment also enhances placental vascular function and materno‐foetal nutrient transfer when given at later stages of pregnancy. Dietary arginine supplementation towards the end of pregnancy thus increased birth weight of piglets (Wu et al., 2012) and reduced intra‐uterine growth retardation in ewes with multiple foetuses (Lassala et al., 2011). Selected clinical trials have been performed assessing the use of arginine to enhance foetal growth and survival in women. Administration of arginine to women with an IUGR foetus reduced placental apoptosis and improved foetal growth and development. Arginine treatment also decreased the incidence of small for gestational age newborns (Chen, Gong, Chen, Luo, & Xiuquan, 2016; Shen & Hua, 2011). In addition, maternal plasma arginine concentrations were lower in pregnancies with foetal growth restriction and arginine concentration in amniotic fluid from gestational weeks 13 to 15 was positively correlated with birth weight and length and head circumference of the human neonate (Bjørke‐Jenssen, Ueland, & Bjørke‐Monsen, 2017). Intra‐uterine growth retardation and the birth of foals too small for their gestational age are also a well‐known phenomenon in horses (Rossdale & Ousey, 2002). Effects of arginine treatment in mares with an IUGR foetus have not been investigated so far, but arginine supplementation during the last three weeks of pregnancy increased uterine blood flow before and after parturition (Mortensen et al., 2011). Based on research in other species including humans, controlled studies on arginine supplementation of mares suspected to carry a growth‐retarded foetus are thus justified.

Mares that had received an arginine‐supplemented diet in early gestation showed a more pronounced weight increase during the last three months of pregnancy. This increase was not due to differences in foal weight between groups. Arginine supplementation affects the maternal metabolism. When fed throughout gestation and lactation, it enhanced the lactation performance of sows in one study (Mateo, Wu, Moon, Carroll, & Kim, 2008) and affected milk composition but did not change milk yield in another study (Krogh, Oksbjerg, Purup, Ramaekers, & Theil, 2016). In cattle, repeated arginine infusion during late pregnancy increased concentrations of prolactin, growth hormone and insulin concentration in maternal plasma and tended to increase milk yield (Chew, Eisenman, & Tanaka, 1984). Our study may not allow the conclusion that dietary arginine supplementation in early pregnancy affects mare growth in late pregnancy. Throughout the study, however, all mares were kept in one group and except for the arginine supplement from days 14 to 45 of pregnancy were fed identically.

In conclusion, dietary supplementation with L‐arginine in early pregnancy may enhance growth of the equine conceptus at the beginning of placentation but was without effect on weight and size of the foal or placenta at birth.

## CONFLICT OF INTEREST

None of the authors have any conflict of interest to declare.

## AUTHOR CONTRIBUTIONS

CA, MK and JZ designed the project. JZ designed and provided the L‐arginine pellets. MK, WM, EB and CN performed all animal experimentation and collected the animal data. CG and JA performed the endocrine analyses. CA and JA analysed the data. JA and CA drafted the manuscript. All authors read, amended and approved the submitted version of the manuscript.
